# Supplementary material for: The m6A-Related Long Noncoding RNA Signature Predicts Prognosis and Indicates Tumor Immune Infiltration in Ovarian Cancer
Source: Cancers (Basel). 2022 Aug 22;14(16):4056. doi: 10.3390/cancers14164056 (PMC9406778; doi:10.3390/cancers14164056)
Supplement: Supplementary file 1 [file cancers-14-04056-s001.zip › cancers-1826530-supplementary.pdf]

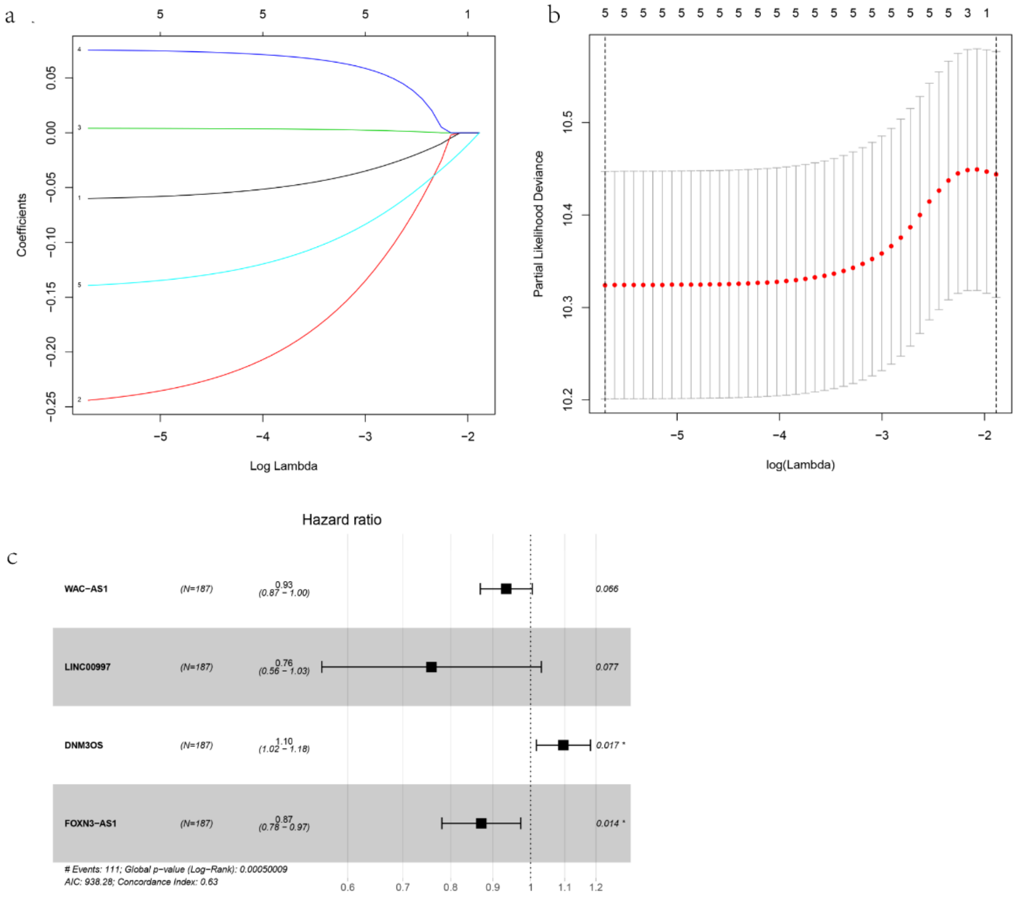

Figure S1 Construction of a risk signature based on m6A-related prognostic lncRNAs in the TCGA-train set.

LASSO regression and multivariate Cox regression were performed, calculating the minimum criteria (a-b) and coefficients (c).

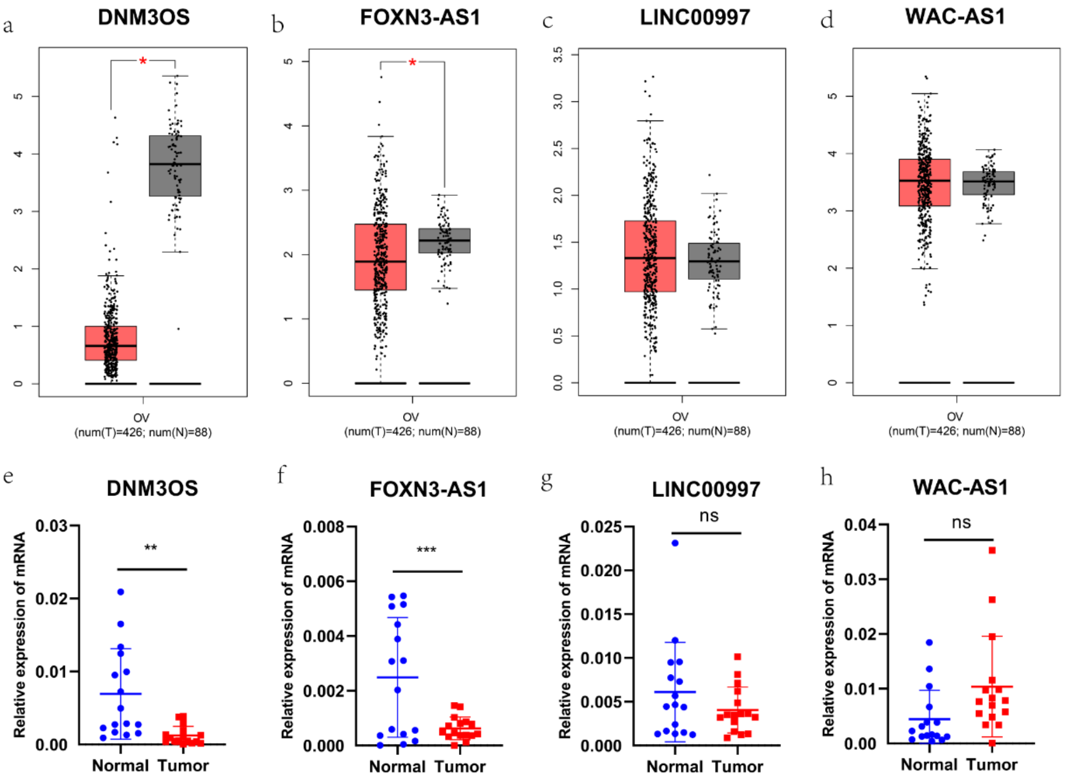

Figure S2 The expression of the four prognostic m6A-related lncRNAs in TCGA dataset and our own samples.

(a-d) The expression of the four prognostic m6A-related lncRNAs in tumor (N=426) and normal tissue (N=88) in TCGA dataset. (e-h) The expression levels of the four prognostic m6A-related lncRNAs mRNA in normal (N=16) and tumor (N=16) clinical tissue samples. *P < 0.05, **P < 0.01, and ***P < 0.001.

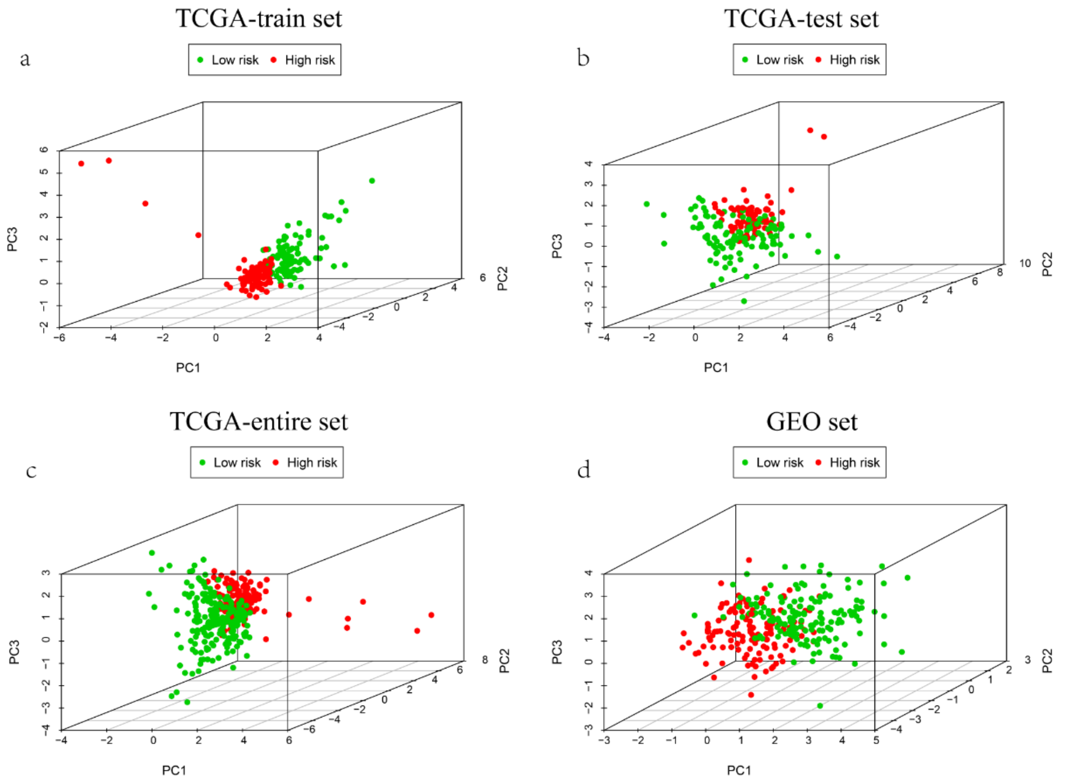

Figure S3 Principal component analysis between the high- and low-risk groups based on the m6A-related lncRNA prognostic signature in the TCGA-train (a, N=187), TCGA-test (b, N=187), TCGA-entire (c, N=374) and GEO (d, N=278) dataset.

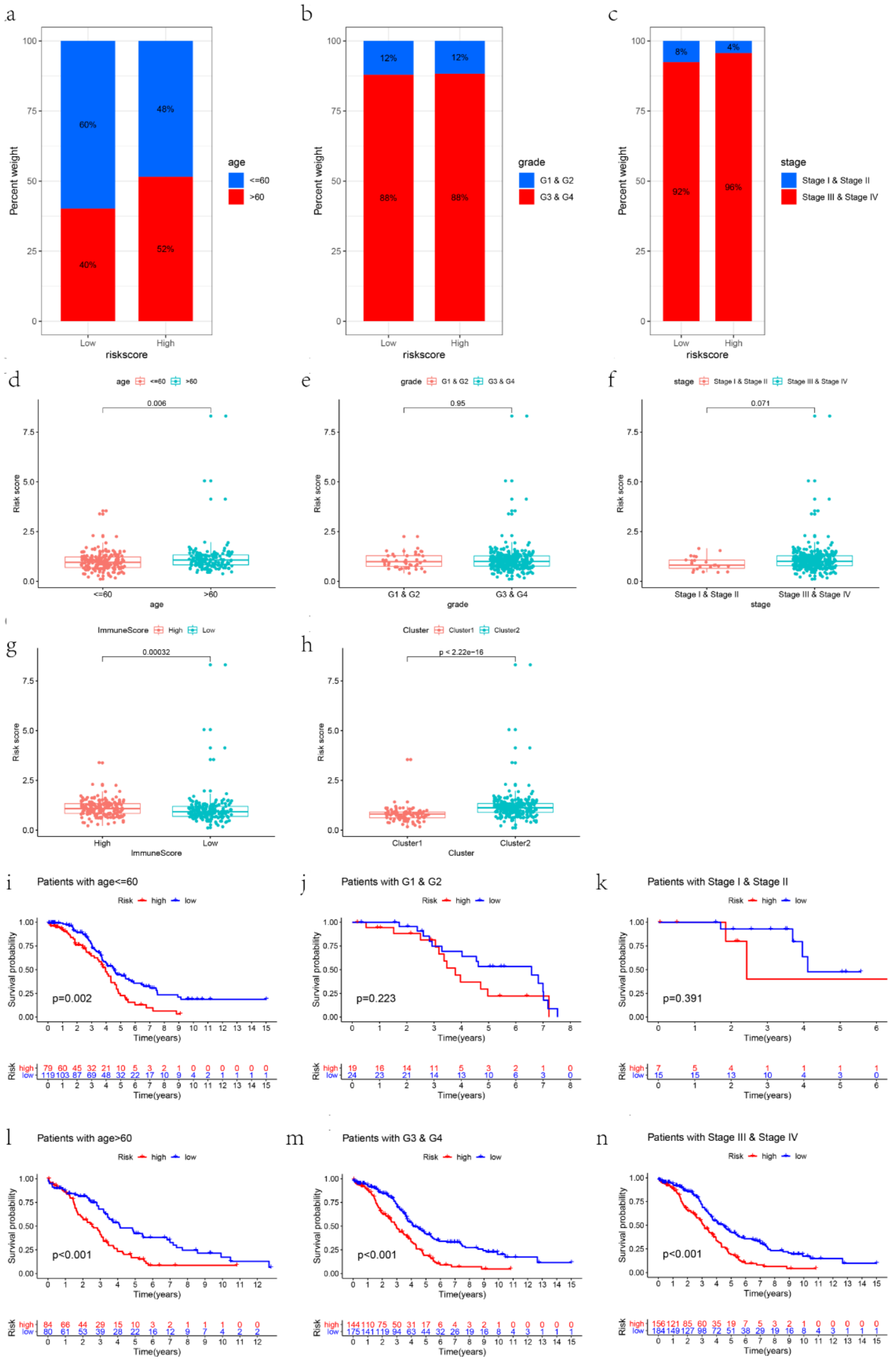

Figure S4 Risk Scores Correlated with Clinicopathological Features in TCGA train set. (a-c) The proportion of patients with different clinicopathologic features in high- and low-risk groups. (d-h) Risk scores in groups stratified by patient age, tumor grade, tumor stage, immune score and consensus cluster. (i-n) The Kaplan–Meier curve for patients with different risk score in subgroups stratified by clinical features.

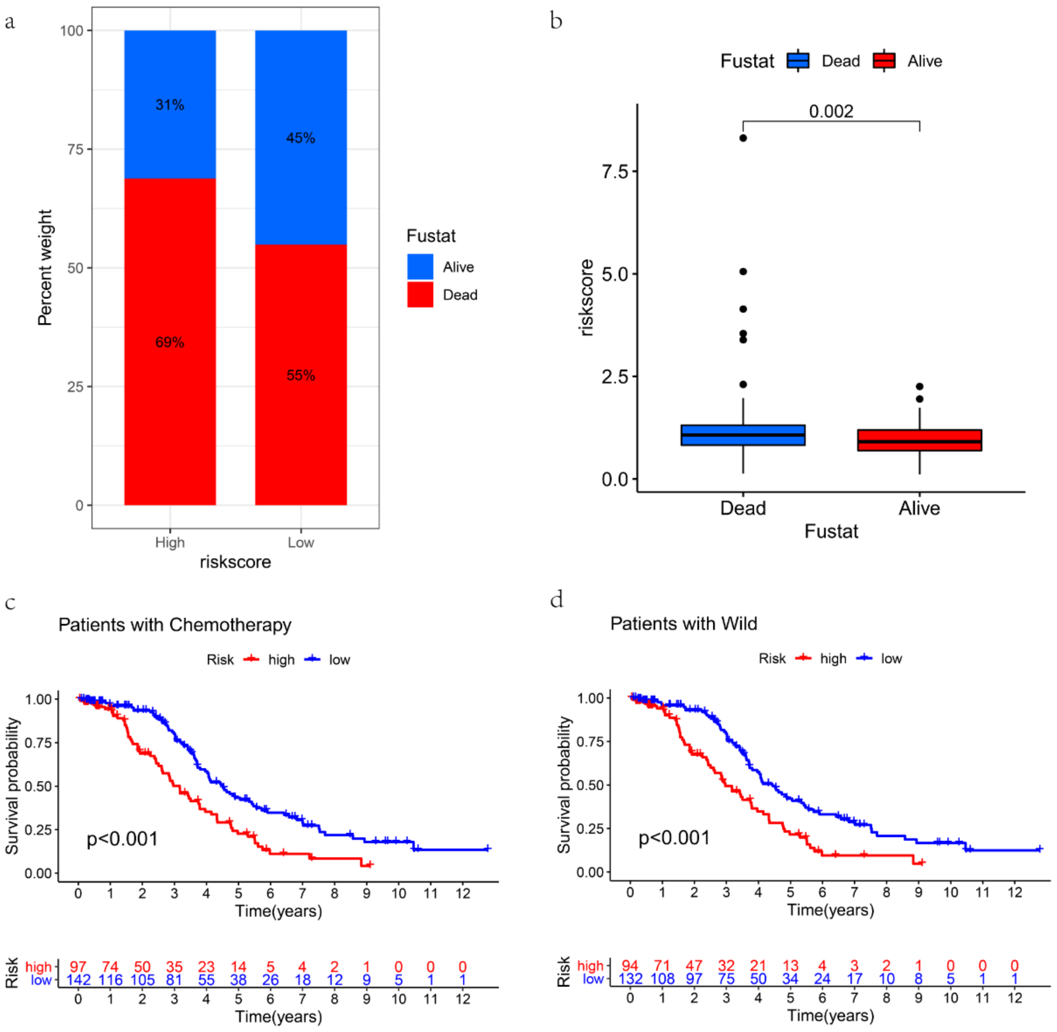

Figure S5 Risk score correlated with other clinical features.

(a) The proportion of patients' state in high- and low-risk groups. (b) The risk score of patients with different state. (c) Kaplan-Meier curves of OS for patients with chemotherapy. (d) Kaplan-Meier curves of OS for patients with BRCA1.

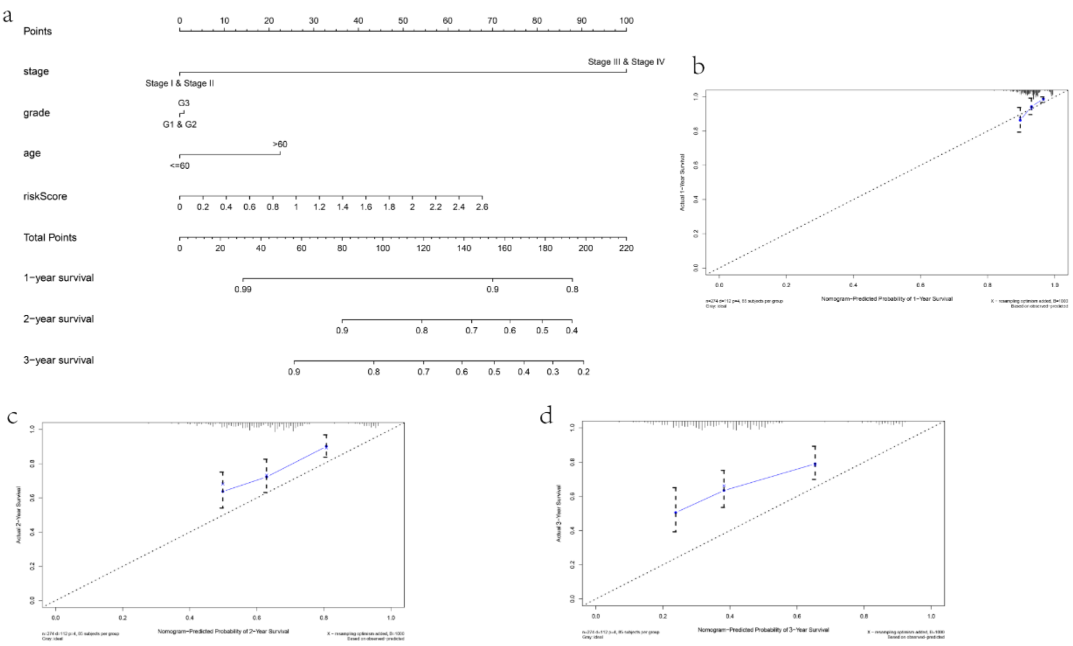

Figure S6 A nomogram for OV patients in GEO dataset.
(a) A nomogram for predicting the 1-, 2-, and 3-year OS of OV patients. (b-d) Calibration curves for the prediction of 1-, 2- or 3-year overall survival of OV patients.

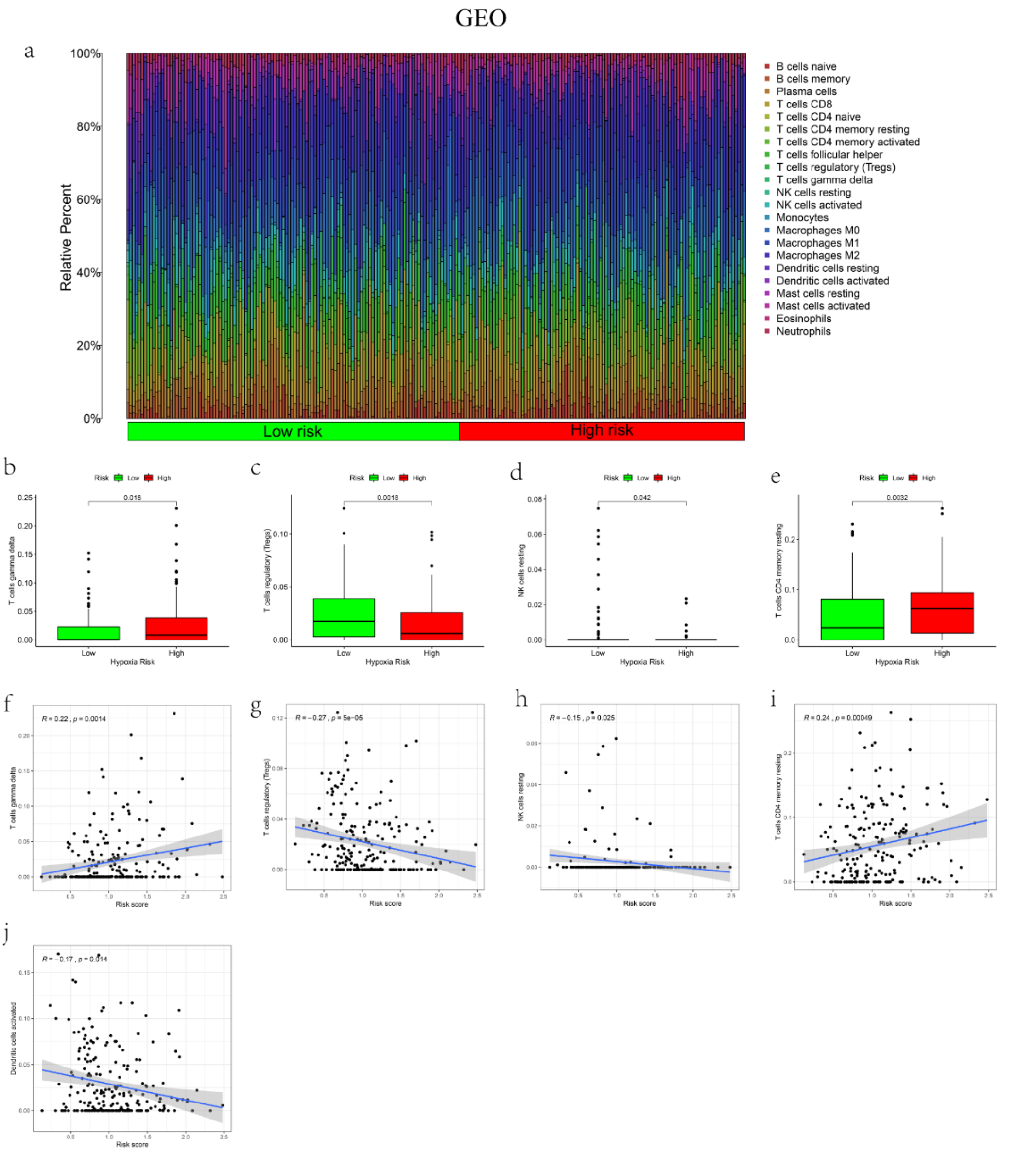

Figure S7 Relationships between the risk score and immune cell infiltration in GEO dataset.

(a) The proportion of 22 immune cells infiltration in high- and low-risk groups. (b-i) Correlation of expression of the m6A-related lncRNAs and infiltration of specific immune cell type.

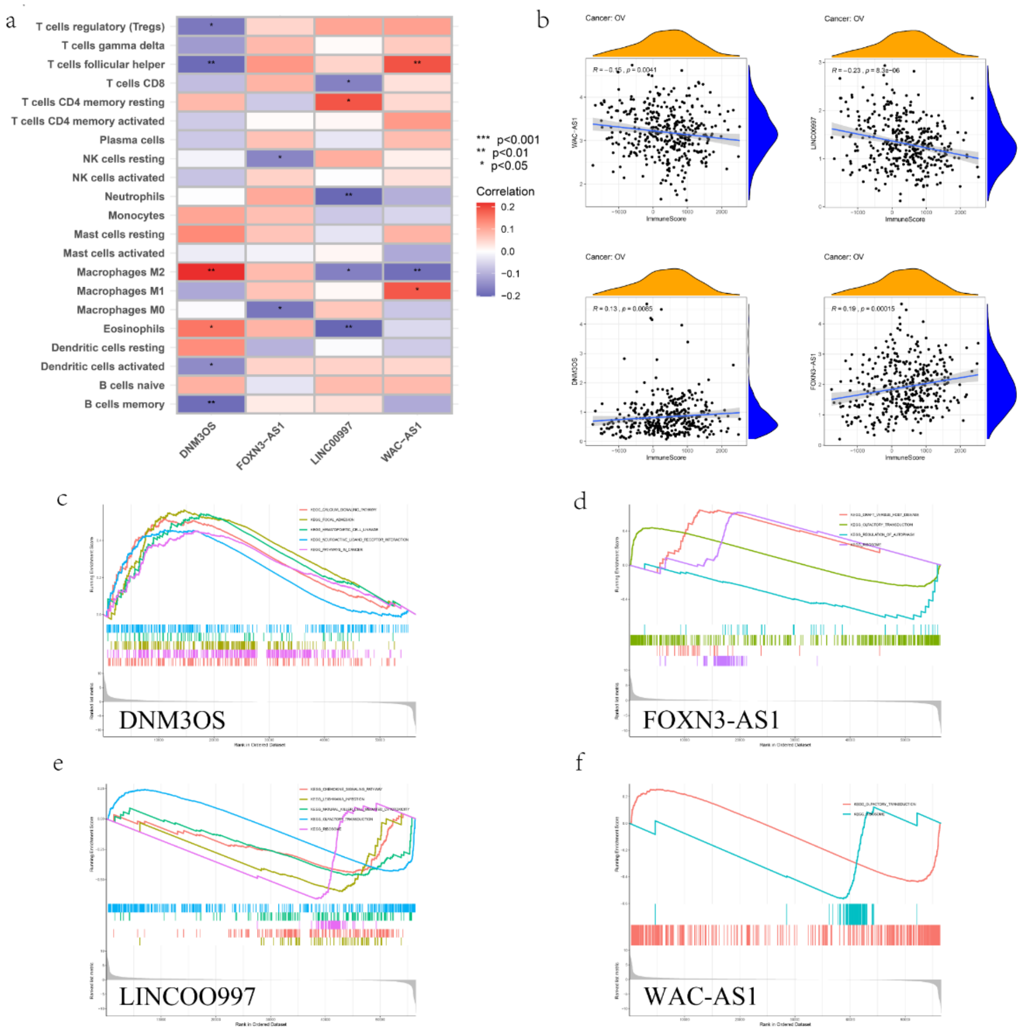

Figure S8 Association of expression of the m6A-related lncRNAs with immune features.

(a) The heatmap of correlation between the m6A-related lncRNAs and immune infiltrated cells. (b) The correlation between the m6A-related lncRNAs and immune score. (c-f) GSEA analysis indicating the most enriched pathways of the m6A-related lncRNAs in OV.

Spearman correlation analysis was used to calculate the association.

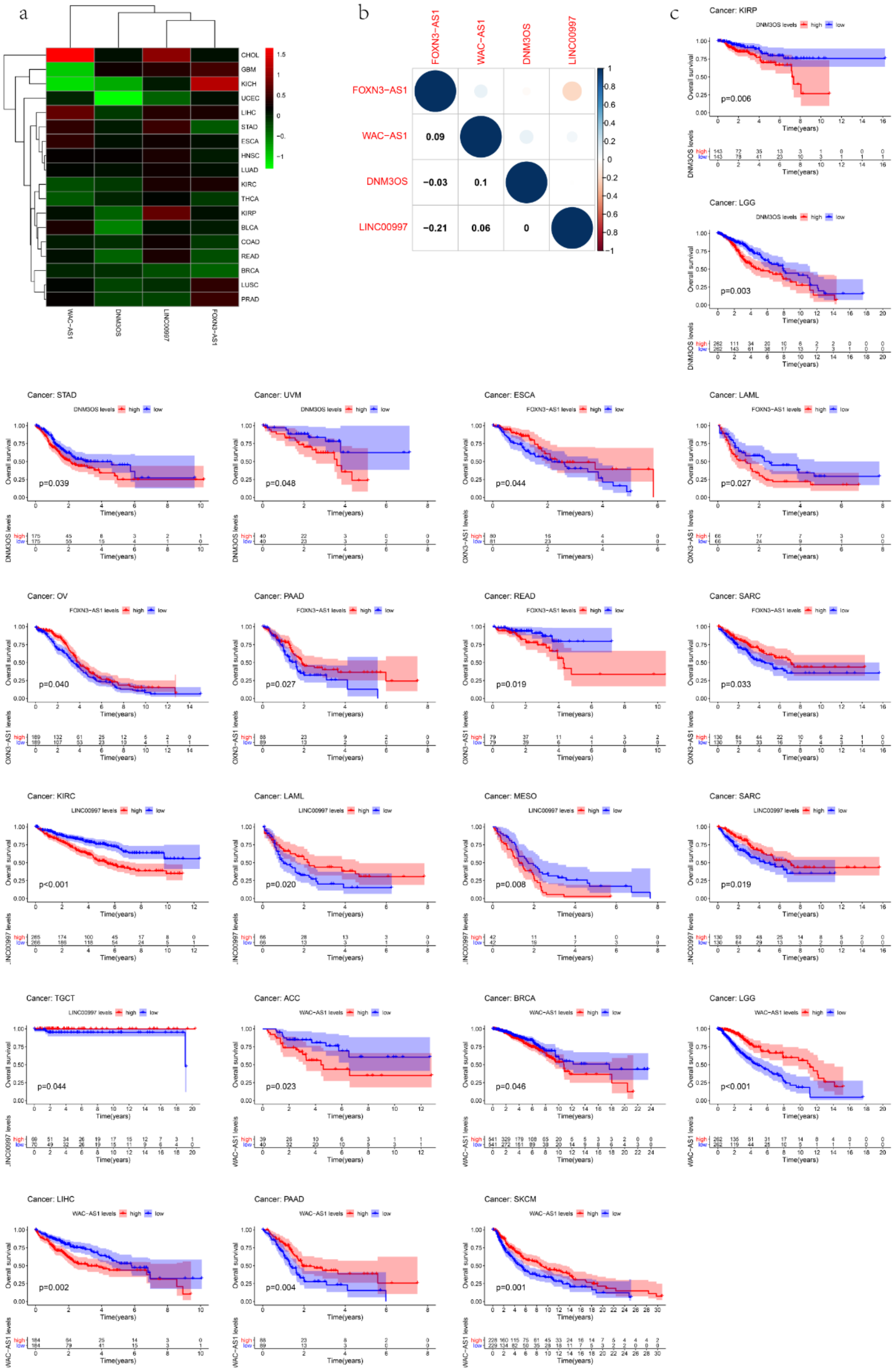

Figure S9 Expression levels of 4 m6A-related lncRNAs in cancers and adjacent normal tissues.

(a) Heatmap showing the expression level of the m6A-related lncRNAs in tumor

tissue compared with normal tissue in 18 cancer types which were composed of more than 5 normal samples. (b) Correlation plot based on Spearman Correlation analysis showing the correlation of gene expression among the 4 m6A-related lncRNAs across all 33 cancer types. (c) The association of 4 m6A-related lncRNAs with patients' prognoses in pan-cancer. The representative top 2 cancer types among the m6A-related lncRNAs are shown according to the p-value.

a

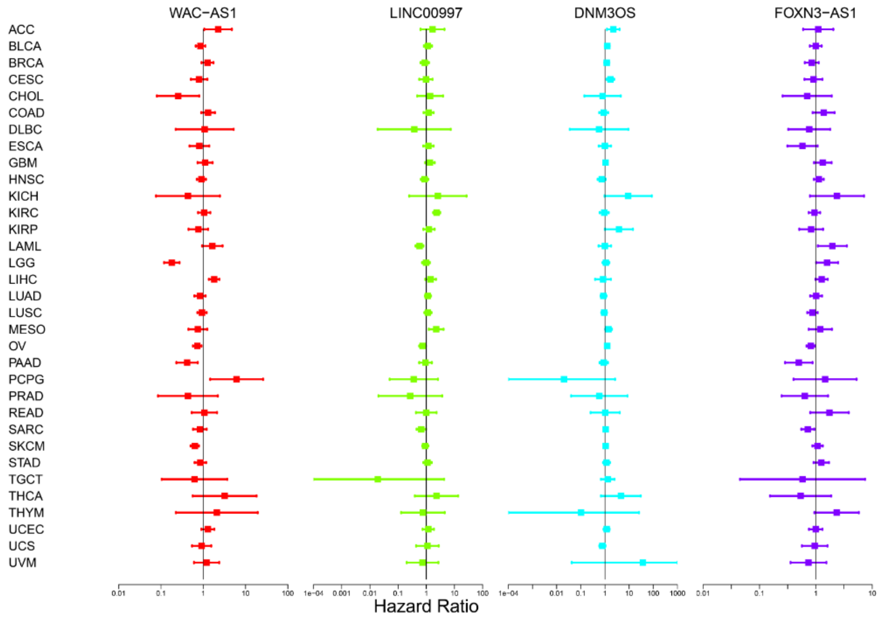

b

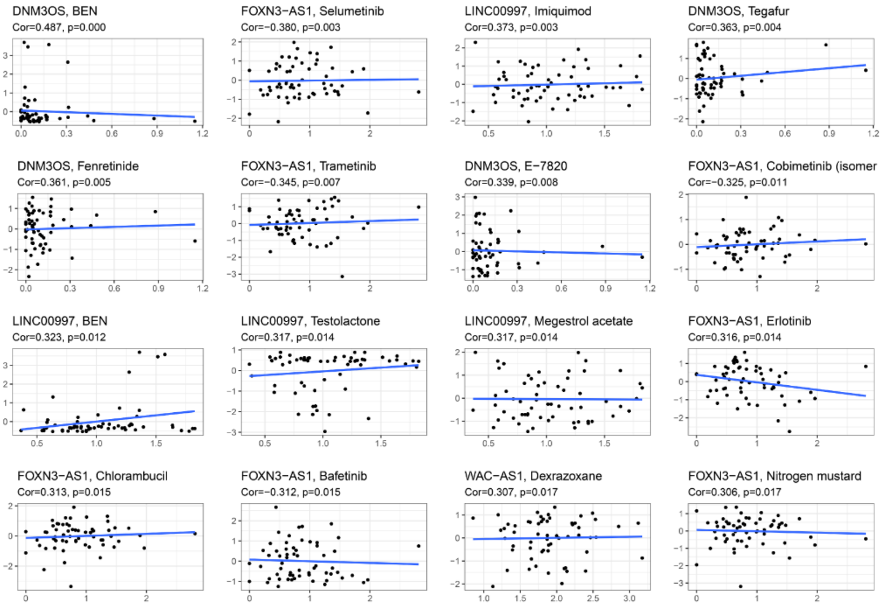

Figure S10 Association of expression of the m6A-related lncRNAs with patients' prognoses and drug sensitivity in pan-cancer.

(a) The forest plots with 95% confidence intervals and hazard ratios for overall survival for different cancer types in pan-cancer. (b) The scatter plot of the correlation between the m6A-related lncRNAs expression and drug sensitivity (the Z score of the CellMiner interface) for the Pearson correlation test using NCI-60 cell line data. Top 16 associations are shown, ordered by p-value.

Table S1. Primers' information.

Gene	Forward primer	Reverse primer
WAC-AS1	5'CAGTGAAGTGGAAGCATGTGTT3'	5'TTCTTTGACCCTTCCTTTCCAG3'
LINC00997	5'ATTCCCTAGCATTGCAGCCT3'	5'TGCTCAGCGATTTCTACCCT3'
DNM3OS	5'GTCCTAAATTCATTGCCAGTTCC3'	5'CACACTCAAGGGCTGTGATTTC3'
FOXN3-AS1	5'TGGGACTTGCTTCCTGACCT3'	5'TGCAGCTTGCTTATTTGTGACT3'
GAPDH	5'ACCACAGTCCATGCCATCAC3'	5'TCTAGACGGCAGG TCAGGTC3'

Table S2. A total of 5 m6A-related prognostic lncRNAs in the TCGA-train dataset

ID	HR	HR.95L	HR.95H	P value
WAC-AS1	0.92695	0.861806	0.997018	0.041323
LINC00997	0.728789	0.537317	0.988491	0.041911
ZFAS1	1.006166	1.000452	1.011913	0.034388
DNM3OS	1.119609	1.041751	1.203287	0.002125
FOXN3-AS1	0.879052	0.791255	0.976591	0.016343

Table S3. The conjoint univariate and multivariate Cox regression analyses of the independent predictor of OV.

univariate Cox regression analysis					multivariate Cox regression analysis				
TCGA-train set	HR	HR.95L	HR.95H	P value	TCGA-train set	HR	HR.95L	HR.95H	P value
Age	1.404	0.959	2.054	0.081	Age	1.292	0.874	1.909	0.198
Stage	7.799	1.087	55.945	0.041	Stage	7.440	1.016	54.504	0.048
Grade	1.128	0.652	1.952	0.667	Grade	0.873	0.500	1.524	0.632
RiskScore	1.492	1.265	1.760	<0.001	RiskScore	1.435	1.205	1.708	<0.001
TCGA-test set	HR	HR.95L	HR.95H	P value	TCGA-test set	HR	HR.95L	HR.95H	P value
Age	1.417	0.978	2.052	0.065	Age	1.407	0.971	2.039	0.071
Stage	0.948	0.384	2.336	0.907	Stage	0.876	0.352	2.180	0.776
Grade	1.293	0.709	2.357	0.402	Grade	1.290	0.703	2.365	0.411
RiskScore	1.497	1.048	2.138	0.027	RiskScore	1.513	1.047	2.187	0.027
TCGA-entire set	HR	HR.95L	HR.95H	P value	TCGA-entire set	HR	HR.95L	HR.95H	P value
Age	1.391	1.068	1.810	0.014	Age	1.327	1.017	1.732	0.037
Stage	2.053	0.911	4.629	0.083	Stage	1.849	0.808	4.230	0.146
Grade	1.189	0.794	1.781	0.401	Grade	1.080	0.715	1.629	0.715
RiskScore	1.487	1.286	1.720	<0.001	RiskScore	1.445	1.246	1.677	<0.001
GEO dataset	HR	HR.95L	HR.95H	P value	GEO dataset	HR	HR.95L	HR.95H	P value
Stage	7.006	2.221	22.098	0.001	Stage	6.768	2.124	21.566	0.001
Grade	1.302	0.882	1.924	0.184	Grade	1.018	0.683	1.517	0.931
Age	1.563	1.074	2.273	0.020	Age	1.536	1.052	2.242	0.026
RiskScore	1.671	1.128	2.476	0.011	RiskScore	1.645	1.093	2.476	0.017
